# Supplementary material for: Antimicrobial Peptide against Mycobacterium Tuberculosis That Activates Autophagy Is an Effective Treatment for Tuberculosis
Source: Pharmaceutics. 2020 Nov 9;12(11):1071. doi: 10.3390/pharmaceutics12111071 (PMC7697726; doi:10.3390/pharmaceutics12111071)
Supplement: Supplementary file 1 [file pharmaceutics-12-01071-s001.pdf]

pharmaceutics
MDPI

Supplementary Materials: Antimicrobial Peptide against *Mycobacterium Tuberculosis* That Activates Autophagy Is an Effective Treatment for Tuberculosis

Erika A. Peláez Coyotl, Jacqueline Barrios Palacios, Gabriel Muciño, Daniel Moreno-Blas, Miguel Costas, Teresa Montiel Montes, Christian Diener, Salvador Uribe-Carvajal, Lourdes Massieu, Susana Castro-Obregón, Octavio Ramos Espinosa, Dulce Mata Espinosa, Jorge Barrios-Payan, Juan Carlos León Contreras, Gerardo Corzo, Rogelio Hernández-Pando and Gabriel Del Rio

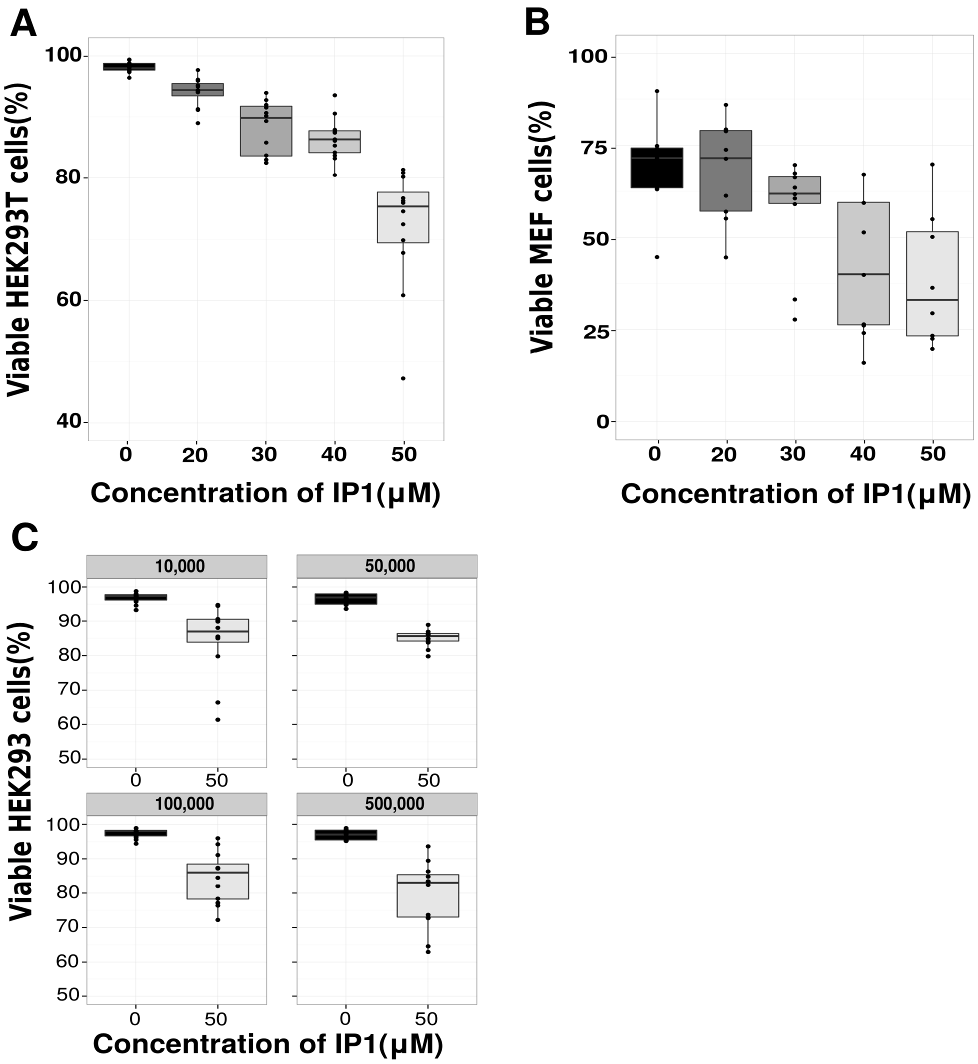

Figure S1. IP-1 cytotoxicity in mammalian cells. HEK293T and MEF cells were incubated with IP-1 and stained with trypan blue to determine viability. As summarized in this figure, IP-1 elicits a cytotoxic effect in both cell lines but is less pronounced in HEK2913T cells A) that in MEF cells B). C) Evaluation of the toxic effect of IP-1 by altering the peptide's interaction with cell membrane, the ratio IP-1/membrane surface was inferred by changing the initial number of HEK293T cells (10,000, 50,000, 100,000 and 500,000) exposed to a constant concentration of IP-1 (50 μM); it is observed that

the cytotoxicity does not change with the number of HEK293T cells. The data presented in these images correspond to at least 6 independent experiments.

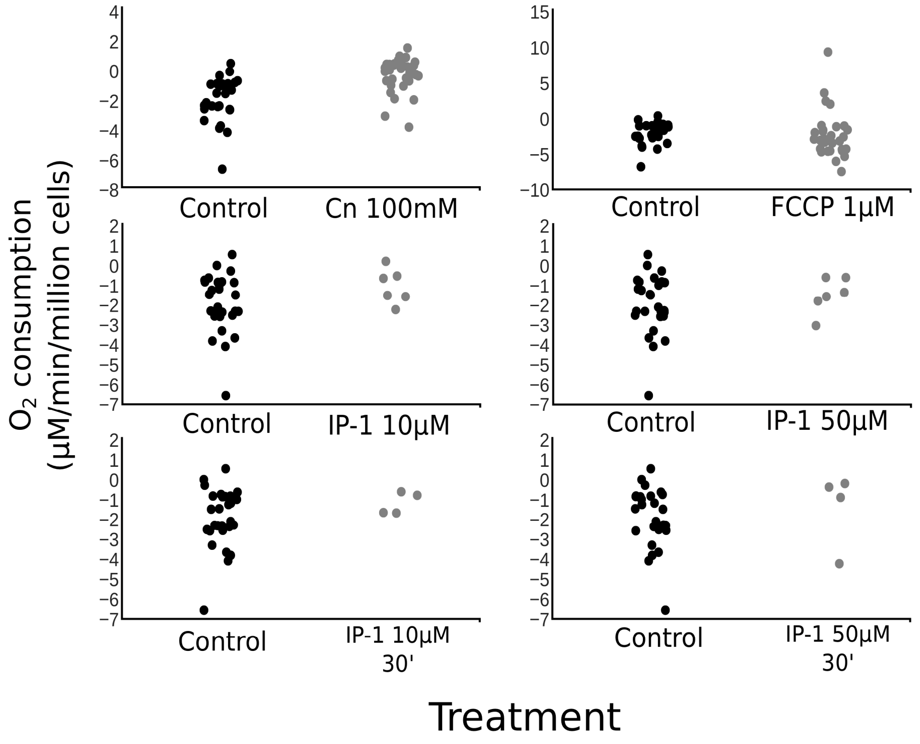

Figure S2. Measuring respiratory effects by IP-1. IP-1 does not induce respiratory arrest in mammalian cells. HEK293T cells were placed in the oroboros high resolution respirometer and treated with either Cyanide 100 mM (respiration inhibitor), FCCP 1 μM (respiration increase) or IP-1 at 10 μM, 50 μM. Each dot represents the highest respiration rate (slope) obtained for each condition. IP-1 does not inhibits or induce respiration in HEK293T cells even after 30 minutes. The image summarizes at least 4 independent experiments.

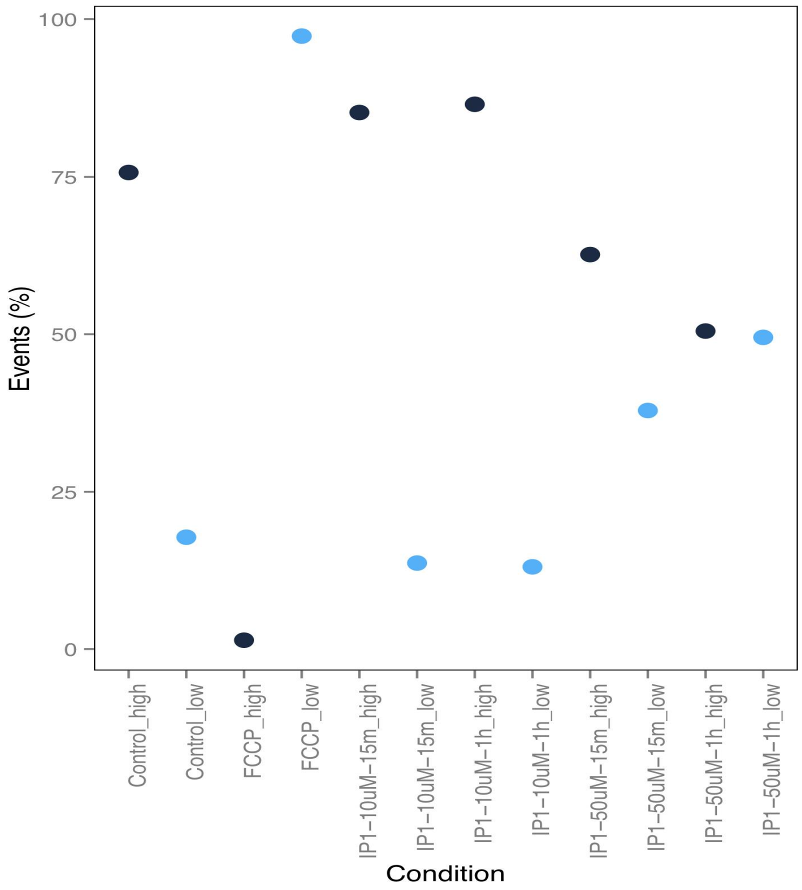

Figure S3. Measuring Mitochondria effects by IP-1. IP-1 does affect mitochondrial membrane potential as measured by following the mitochondrial potential stained by the fluorophore JC-1 (see Methods). The data correspond to >400 to 1500 events/cells analyzed for each condition.

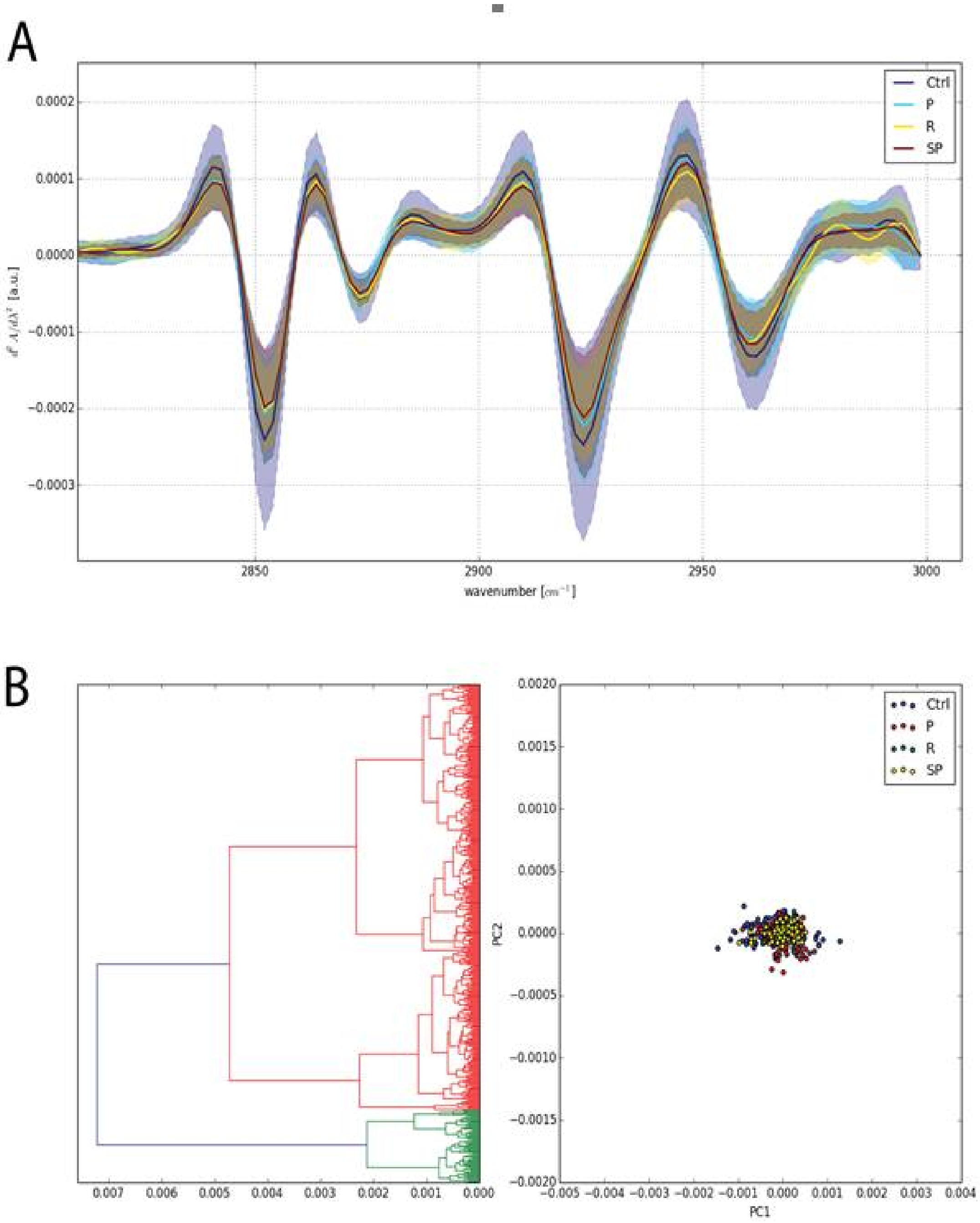

Figure S4. Total lipid content estimation of cells exposed to autophagy inducers**.** A) FTIR spectra of HEK293T cells alone (represented Ctrl in the figure legends) or in the presence of Rapamycin (autophagy inducer; represented by the letter R in the figure legends), Substance P (apoptosis inducer; represented as SP in the figure legends) or IP-1 (represented by the letter P in the figure legends). The peaks representing lipids are no different in any of the treatments and the control. B) hierarchical clustering (3 clusters were identified indicated by the 3 colors, representing protein, lipids and nucleic acids) and C) Principal component analysis of the first 2 components (accounting for 75.9% of the variance in the data) confirm that the characteristics of the spectra are quite similar indicating that the treatments do not alter the content of macromolecules in the cells. The data presented summarizes the spectrum recorded for at least 40 individual cells at each condition.

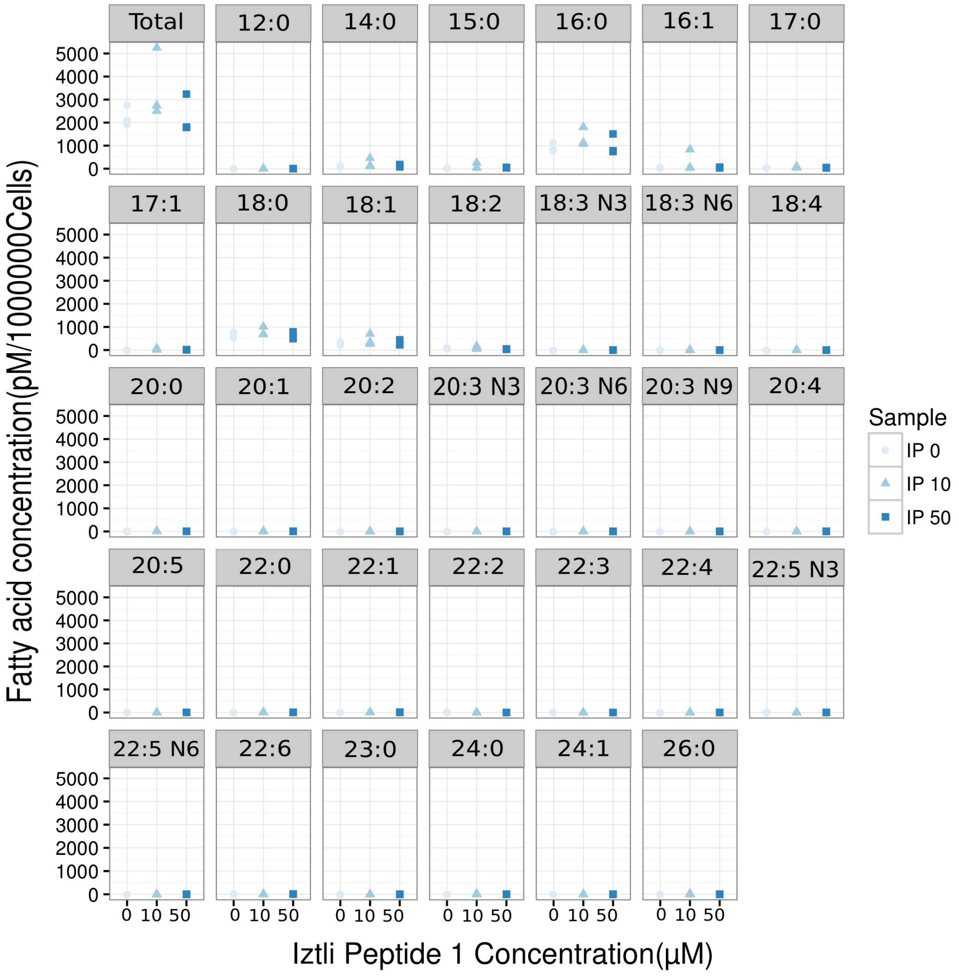

Figure S5. Lipid profile of cells exposed to IP-1. Free (non-esterified) fatty acid concentration of Control (IP 0), IP-1 at 10 μM (IP 10) and 50 μM (IP 50). There is no difference in lipid concentration across each lipid type and the overall lipid concentration as detected by mass spectrospy (see Methods). The fatty panel consists of total fatty acids and 33 individual fatty acids, from C12 to C26 including all saturated fatty acids from 12 to 26 carbons, mono- and poly-unsaturated fatty acids and their positional isomers. The results are presented for 3 independent experiments.

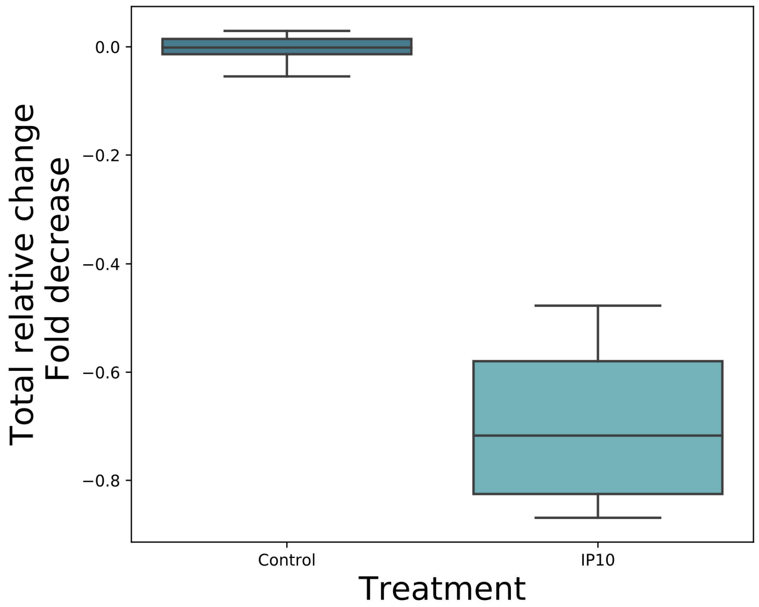

Figure S6. IP-1 inhibits luciferase reaction. Firefly luciferase assay performed to determine whether IP-1 interrupts the interaction between luciferase and ATP. This figure shows how the pre-incubation of ATP with IP-1 10 µM decreases the signal to 50% of the control. The results are presented for 2 independent experiments each with 2 technical duplicates.

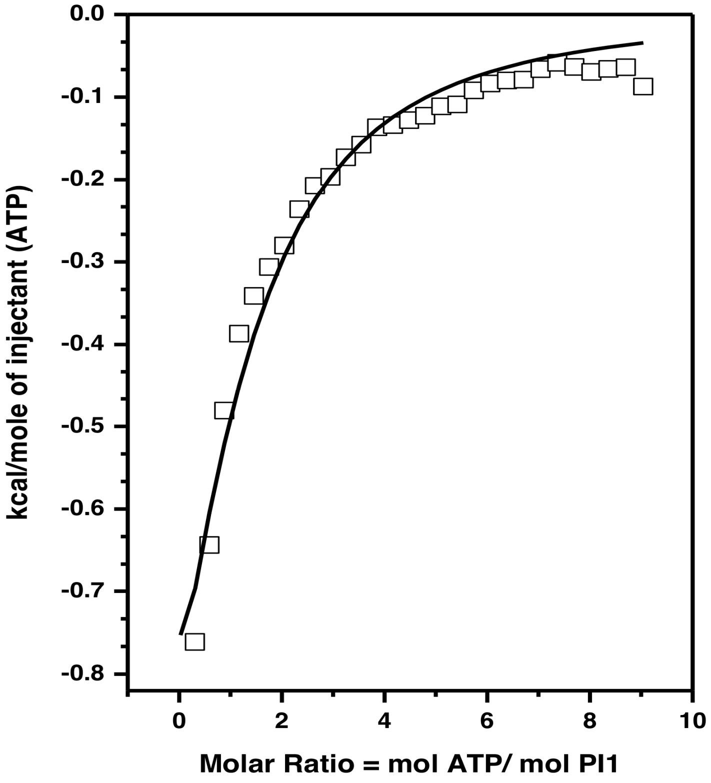

Figure S7. Isothermal Titration Calorimetry binding essay at 25 C. The concentrations used were 10 mM ATP (syringe) AND 0.25 mM PI-1 (cell). The thermodynamic parameters (equilibrium constant and binding enthalpy) for the ATP/PI-1 interaction were obtained using a 1:1 stoichiometry model (continuous line). The image displays 30 independent titrations.

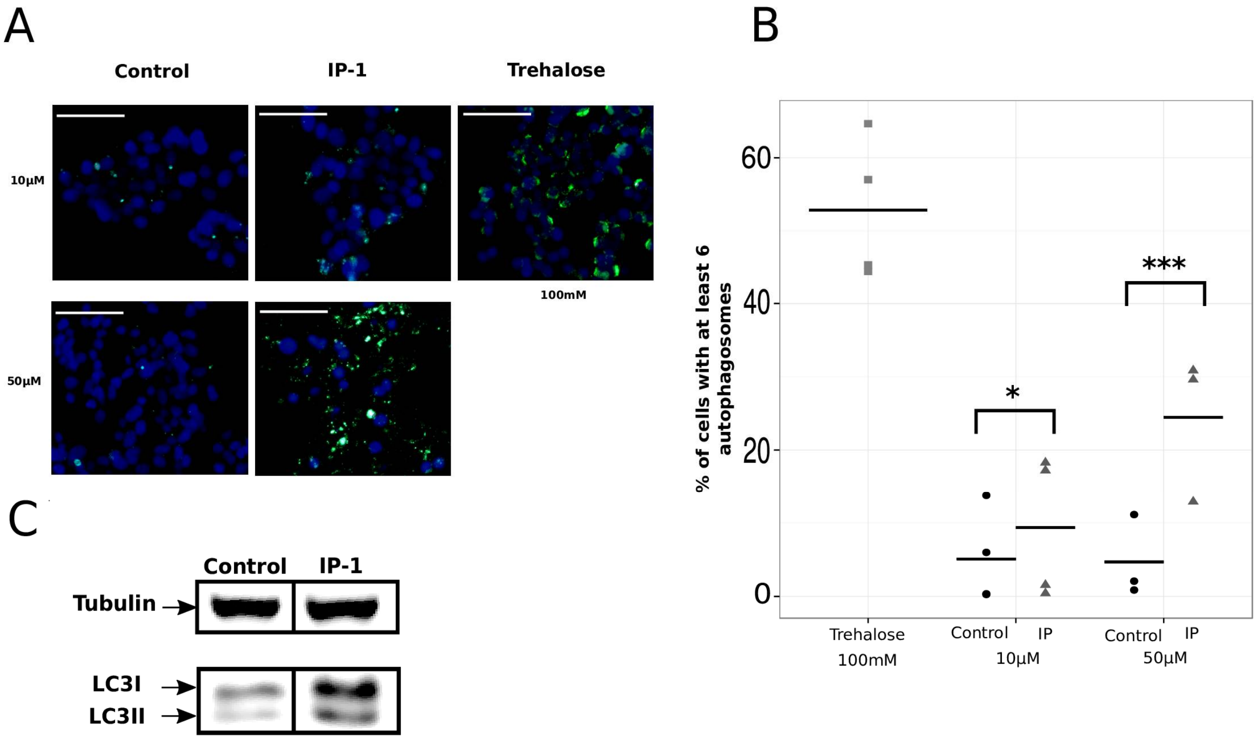

Figure S8. IP-1 induces autophagy in HEK293T cells. HEK293T cells were incubated for 6 h with the indicated concentrations of IP-1 or trehalose (100 mM); as control, cells were exposed to water, the IP-1 solvent. Then they were stained with the Cyto-ID kit to detect autophagosome formation. A) Fluorescence microscopy images show the presence of autophagosomes as green dots in response to Trehalose, a known autophagy inducer, or indicated concentrations of IP-1; the scales bar represent 50 µm. B) Quantification of the cells having at least 6 autophagosomes per cell for at least 3 independent experiments (thousands of cells were analyzed in each experiment); each dot represents an independent experiment. The comparison between samples was conducted using the exact Fisher test; the bar represents the mean values and asterisk represents statistical significance $p<0.05$. C) After incubation, HEK293T cells were lysed and the proteins were extracted to perform a Western Blot to detect LC3, the treatment with IP-1 10 µM induced lipidation of LC3 (indicated as LC3-II), a marker of autophagy induction; the image is representative of 2 independent experiments. LC3-II was detected in Western blots to confirm autophagy activation. Unlipidated LC3-I is a protein that localizes to the cytoplasm in a resting state, but upon autophagy induction it is lipidated on the membrane of the forming autophagosomes, and is then coined LC3-II. LC3-I and LC3-II proteins have different migration patterns in eletrophoretic gels.

The accumulation of autophagosomes in cells may result by an induction of autophagy or a blockage of the autophagosome-lysosome fusion preventing the formation of autolysosomes (autophagic flux inhibition). To distinguish autophagy induction from autophagic flux inhibition, constructs of LC3 fused to GFP or pmRFP were used, since these fluorescent proteins allowed us to identify autolysosomes. When LC3-GFP or LC3-RFP are located in the autophagosome both proteins fluoresce, detecting a yellow color. In contrast, when autophagosomes fuse with lysosomes creating autolysosomes, which are highly acidic organelles, the green fluorescence is quenched and only the red fluorescence remains. Therefore, if the autophagic flux is functional during IP-1 treatment, autolysosomes should be observed as red dots, lacking green flourescence. As shown in Figure S9A, IP-1 is indeed an autophagy inducer and not an autophagy blocker in HEK2913T cells, as red autolysosomes were found. This figure also shows the presence of yellow (red arrow) and red (white arrow) autophagosomes, providing evidence that both proteins were located at the autophagosome. An alternative method to monitor autophagic flux is by following the degradation

of the autophagy receptor p62/SQSTM1. Indeed, p62/SQST1 protein was degraded in cells exposed to 10 µM of IP-1 in HEK293T cells (Figure S9B). Finally, Chloroquine inhibition of autophagosome-lysosome fusion was used to distinguish the blocking from the induction activity of autophagy by IP-1. If the increase of autophagosomes we observed in response to IP-1 treatment (Figure S8A) is due to an inhibition of the autophagic flux, further inhibition with Chloroquine should show no difference. Again, we found evidence of autophagy activation by IP-1 in HEK293T cells, since we observed an accumulation of autophagosomes in the presence of both IP-1 and Chloroquine compared with Chloroquine alone, similarly to that observed by the known inducer of autophagy trehalose (Figure S9C).

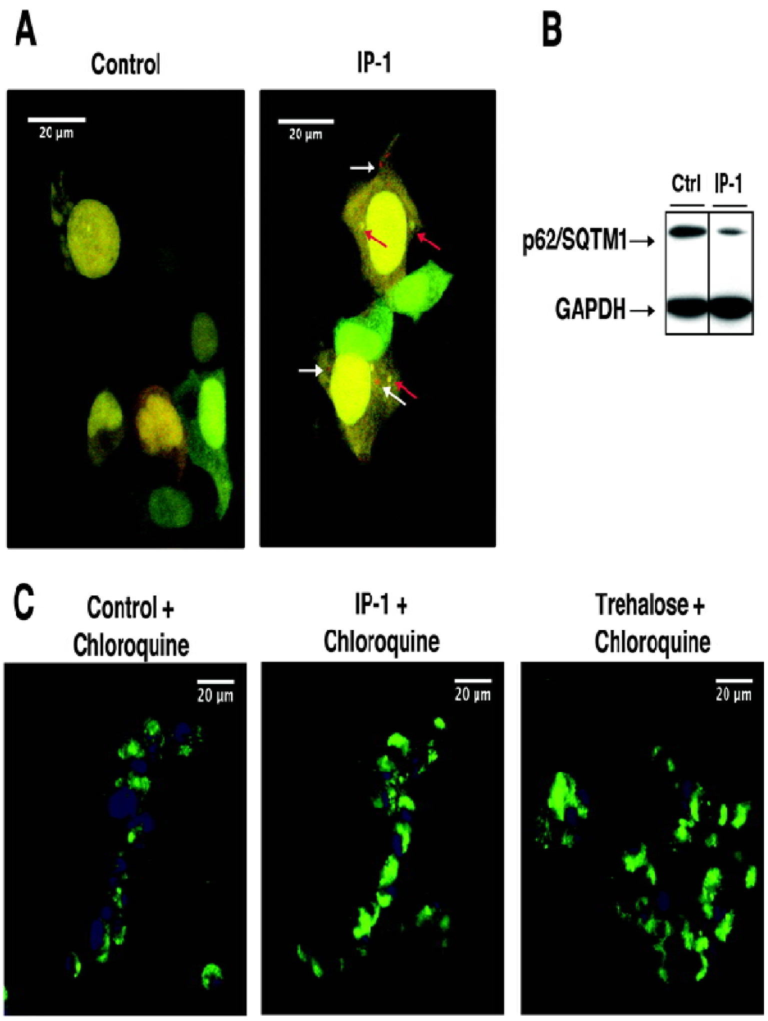

Figure S9. IP-1 induces autophagic fux in HEK293T cells. A) HEK293T cells were transfected with DNA vectors coding for LC3 protein tagged with green or red fuorescent proteins, incubated with IP-1 and a time lapse of confocal microscopy was performed. This panel shows the presence of autophagosomes (yellow dots indicated by red arrows) as well as autolysosomes (red dots indicated by white arrows), indicating that autophagy induced by IP-1 has an uninterrupted fux. B) Western Blot to detect P62/SQSTM1, a protein associated with the autophagosome that is degraded after autolysosome formation; on average, p62/SQTM1 was reduced 13% (3h) and 85% (6h) with respect to the control (estimation was performed using Fiji). A representative blot from 2 independent experiments is shown. C) Control cells treated with Chloroquine 40 µM, a blocker of the autophagosome-lysosome fusion, showed a number of cells with autophagosomes smaller than cells exposed to chloroquine plus IP-1 as shown by staining with Cyto-ID (autophagosomes shown by dense green bodies) or than with Trehalose (a known inducer of autophagy), indicating that IP-1 indeed induces a full fedged autophagic process; these experiments were conducted twice and at least 3 images were recorded for each experiment; the presented image is representative.

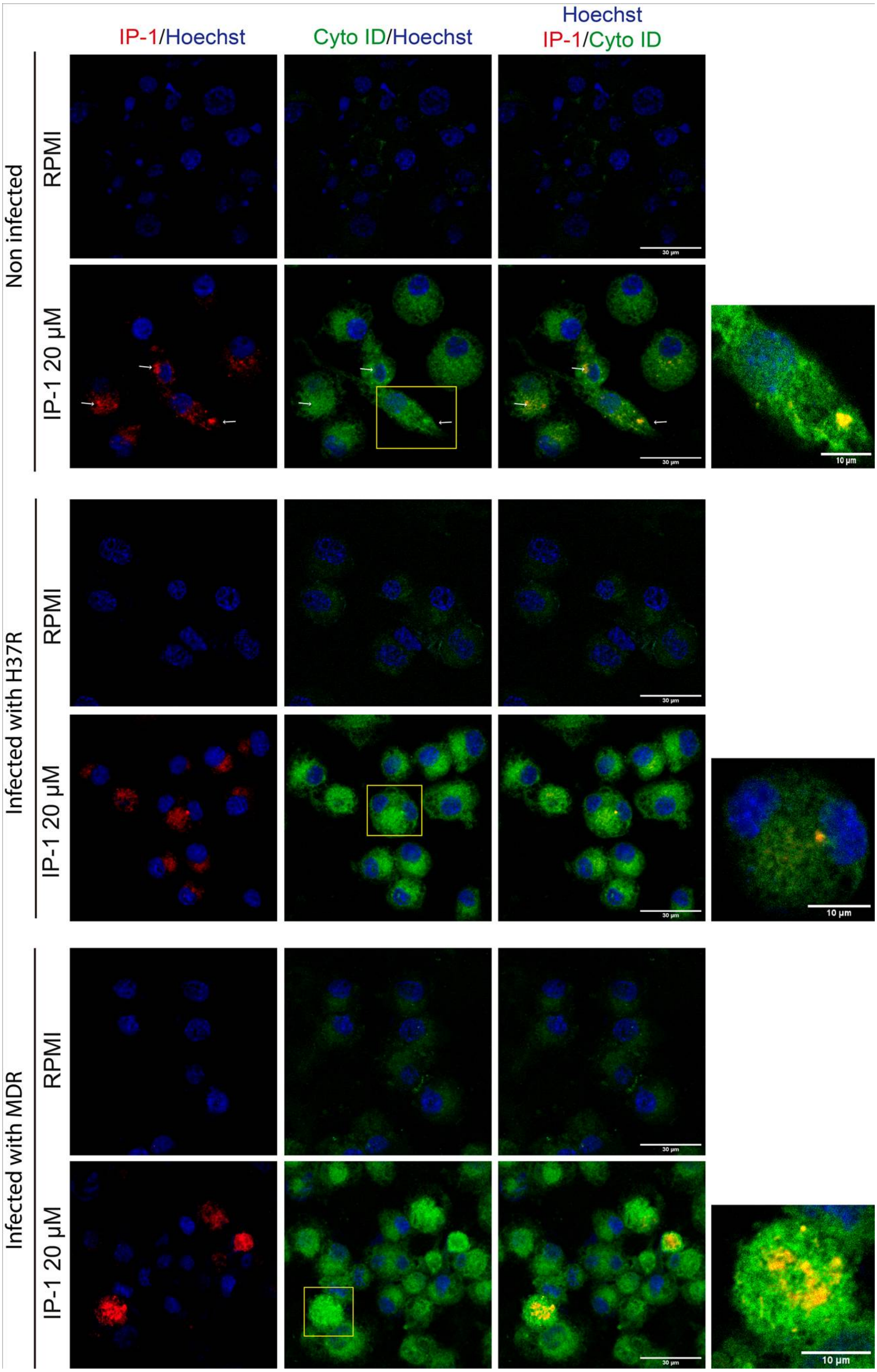
IP-1/Hoechst
Cyto ID/Hoechst
Hoechst
IP-1/Cyto ID
Non infected
Infected with H37R
Infected with MDR
RPMI
IP-1 20 µM
30 µm
10 µm

Figure S10. IP-1 is found in autophagosomes. Macrophages (J774.1) were infected or not with the indicated MBT strains. H37Rv is sensitive to the first line of antibiotics and MDR is resistant to those antibiotics. Autophagosomes were stained with CytoID and the localization of IP-1 was followed using an IP-1 peptide conjugated to the red fluorophore TAMRA Notice that both MBT strains induce autophagy, as expected, although to a lesser extent than when IP-1 is added. TAMRA-IP-1 was found inside autophagosomes (arrows), suggesting that it can reach MBT inside the cell. Representative images acquired by confocal microscopy from three experiments are shown. Yellow squares indicate amplified areas shown at the right, where only one focal plane is shown to demonstrate co-localization of the red signal from IP-1 and the green signal from CytoID (yellow dots).

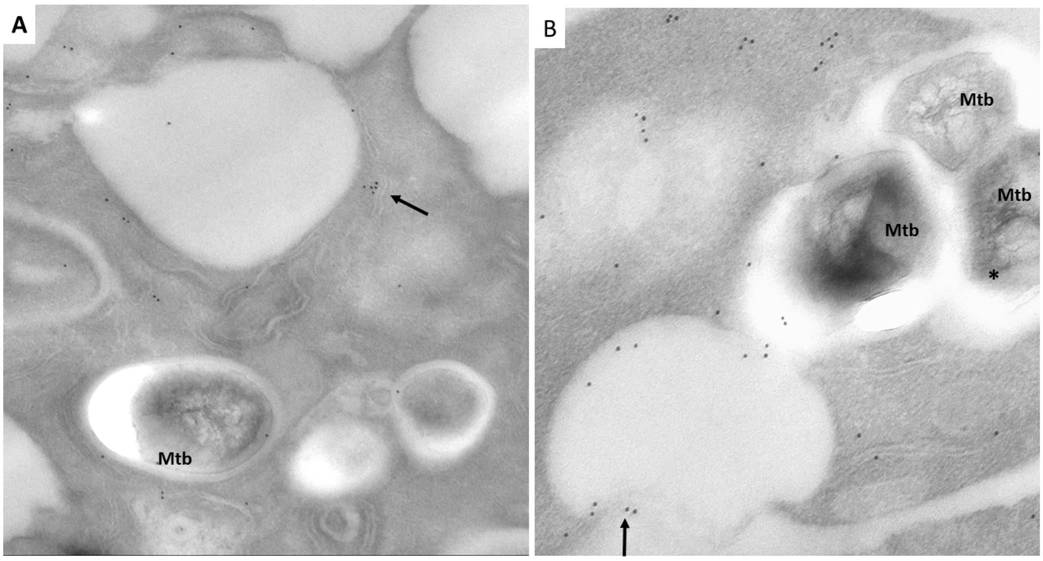

Figure S11. Immunoelectronmicroscopy detection of LC-3. Representative micrographs of subcellular detection of autophagosomes by immunolabeling with specific antibodies against LC3. A) Macrophage after 24 hour of infection showing a phagosome with bacteria (B) near to autophagosome vacuoles surrounded by LC-3 detected by gold labeled antibodies (black dots, arrow). (B) Infected macrophage incubated with LC-3 showing bacteria into phagosomes (B) near to several LC-3 immunogold labeled vacuoles that correspond to autophagosomes (asterisks).

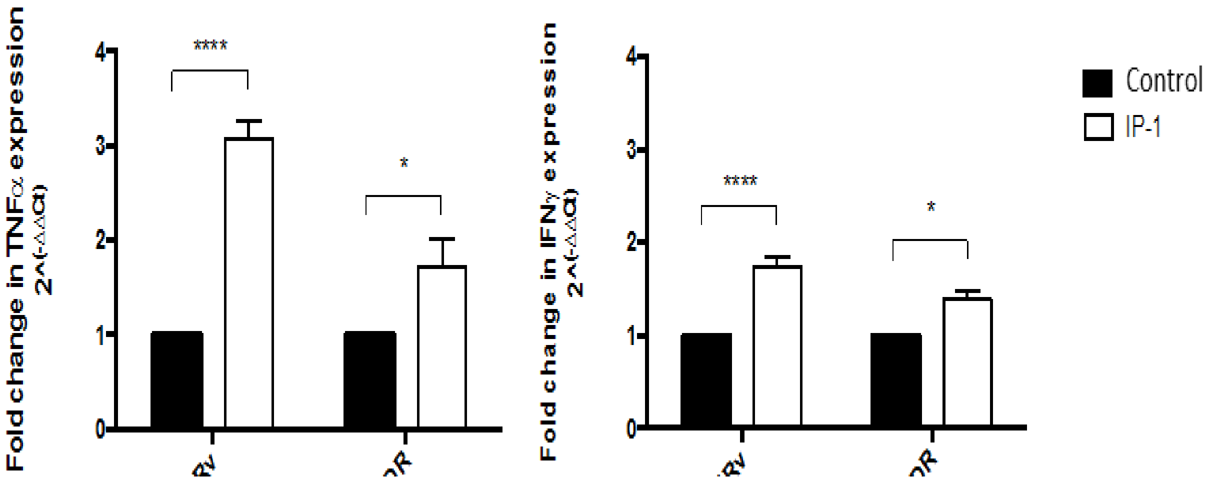

Figure S12. Relative mRNA expression of proinflammatory cytokines TNFα and IFNγ from lung homogenates of MTB-infected mice (H37Rv or MDR) and treated with IP-1. Data are expressed as means ± SD of three different animals after 28-days of treatment. Asterisks represent statistical.
